# Repeated Cross-Sectional Survey Study of Pain Management in Portuguese Pediatric Emergency Departments (2007-2018)

**DOI:** 10.7759/cureus.83042

**Published:** 2025-04-26

**Authors:** Joao P Valente, Andre Garrido, Ricardo Araujo, Clara Abadesso

**Affiliations:** 1 Pediatric Service, Child and Youth Department, Unidade Local de Saúde Amadora/Sintra - Hospital Professor Doutor Fernando Fonseca, EPE, Lisbon, PRT; 2 Research Department, Instituto de Plasmas e Fusão Nuclear and Centro de Recursos Naturais e Ambiente (CERENA) Instituto Superior Técnico, Universidade de Lisboa, Lisbon, PRT

**Keywords:** health care survey, pain, pain management, pediatric emergency medicine, pediatrics

## Abstract

Objectives

The prevalence of pediatric pain, either related to the child’s hospital visit or because of diagnostic and/or therapeutical interventions, is of primordial importance in pediatric emergency departments (PEDs). In this study, we evaluate the evolution of pain assessment and management in Portuguese PEDs over 11 years.

Methods

We prepared a questionnaire addressed to head physicians of 45 Portuguese PEDs in 2007 and statistically compared the responses to those provided in 2018, where we also posed these questions to nurse managers.

Results

Pain assessment in our cohort of Portuguese PEDs has significantly improved, namely, with the establishment of local protocols and widespread use of pain scales (from 52% to 93%, p = 0.03). However, effective adoption of pain management remains insufficient, as mild to moderate pain is still far from being universally treated (only 22% always use analgesia). Nonetheless, there seems to be adequate treatment of severe pain and respective common use of opioids, but correct practices were not generally adopted when specific types of pain were analyzed. Procedural sedation and analgesia have significantly increased but are not yet universally practiced. In the year 2018, about 88% of these inadequacies are reflected by the staff’s perception that pain management remains suboptimal, and more training is needed.

Conclusion

The development of collective awareness and the institution of national guidelines for pediatric pain have helped to improve the conjuncture in Portuguese PEDs. However, a coordinated nationwide plan to promote local training and optimize knowledge translation is still lacking.

## Introduction

In recent decades, the recognition of pain relief as a fundamental right has resulted in significant progress in understanding and assessing pediatric pain. Indeed, children experience pain distinctively from adults, as they exhibit differences in the neurobiology of pain [1]. Pain is a chief complaint in the pediatric emergency department (PED), being prevalent in more than 60% of the cases at admission [2,3]. In this context, pain must be promptly assessed and treated, regardless of the underlying condition. Despite ongoing improvements in pain relief, pediatric pain management is still insufficient worldwide [4-6]. Ironically, pain induced by health professionals during several routine procedures is often underestimated, being among the most common sources of acute pain at the PED [1,7].

Pain treatment must be central to pediatric action as any form of oligoanalgesia has short- and long-term consequences. In the short term, acute pain heightened pain perception, anxiety, and fear, which prevents accurate physical examination and delays necessary interventions. Consequently, potential needless exacerbation of the child’s condition and the increased length of stay at the PED could be avoided. In the long term, untreated pain may affect a child’s emotional well-being and increase the possibility of chronic pain [6,8,9]. It is, thus, crucial that pain is accurately assessed and that nurse-initiated pain treatment protocols are instituted. Pain treatment must be prioritized and optimized, using non-pharmacological interventions to reduce distress and anxiety, combined with pharmacological treatment, proportional to pain severity [10-12].

Allied to this avoidable scenario is the fact that pediatric pain has only started to receive attention in the 1980s [13]. Portugal established the National Day Against Pain as early as 1999 and created a National Plan Against Pain in 2001 [14], which has been periodically updated [15]. In 2003, pain was considered the “fifth vital sign,” which enforced systematic records of pain intensity [16], and in 2010, pediatric-specific guidelines were published by the Ministry of Health [17]. Since then, specific pediatric guidelines were published covering pain management in neonates, children with cancer, and invasive procedures [18-20]. Despite this favorable context, no studies demonstrate to what degree these recommendations have been converted into local protocols at the PEDs. Also, there is no national data regarding the evolution of pain control over the years, and how it compares with other countries.

Some findings from this article were previously published on the medRxiv preprint server (July 8, 2022) with a different research focus, revised interpretation of results, and analysis of distinct populations, leading to different conclusions and implications for the pediatric pain management literature.

This study aims to describe the policies and reported practices of pain management at a national level by comparing the results between two surveys performed in 2007 and 2018 in a sample of Portuguese PEDs.

## Materials and methods

Study design

In 2007, a structured questionnaire was sent via email to all 45 Portuguese hospitals with PEDs, including both private and public institutions, to be completed by the head physician of each PED. In 2018, a similar questionnaire - this time created using Google Forms (Google, Inc., Mountain View, CA) - was sent to the same 45 hospitals (newly established PEDs were not included; see Appendices). In this iteration, both the head physician and the head nurse of each PED were asked to respond, acknowledging the essential role that nurses play in the assessment and management of pain in children and adolescents, beginning at the point of hospital entry, particularly during the initial triage process. Most questions had a Likert scale: never, <50% of the time, > 50% of the time, and always. Responses to questionnaires were collected during 2007 and between the 1st of August of 2018 and the 31st of July of 2019, respectively. It was not always the same person answering the questionnaire between the two time intervals. We excluded all repeated questionnaires, and we only accepted the original responses when questionnaires were repeated. Most of the questions required an answer to move forward in the 2018 questionnaire. Whenever the 2007 questions remained unanswered, the sample was adjusted accordingly - that is, based on the total number of responses rather than the total number of hospitals. This project was developed by our Pediatric Service from the Child and Youth Department in Unidade Local de Saúde Amadora/Sintra - Hospital Professor Doutor Fernando Fonseca, EPE, Lisbon, Portugal. Ethical approval for this study was obtained from the Ethics Committee for Health of Professor Dr. Fernando Fonseca Hospital, EPE.

Statistical analysis

**Figure 1:**
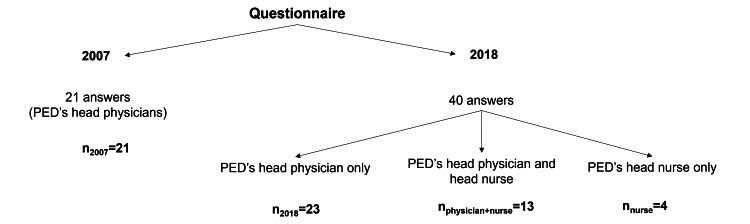
Rationale for the various analyses performed and corresponding sample adaptations

The statistical analysis was conducted using the chi-square (χ²) test to compare the distribution of categorical variables among three independent groups. We employed the chi-square test to evaluate the statistical relationship between survey responses from 2007 and 2018. Given the study's repeated cross-sectional design, comparing responses at two distinct time points, the chi-square test was appropriate for assessing changes in the distribution of categorical data over time. This method is particularly suitable for determining whether the observed differences in approaches to pediatric pain management in emergency departments between the two survey periods were statistically significant. Although the survey responses were based on a Likert scale, providing ordinal data, we treated the responses as categorical variables for analysis. The chi-square test allowed us to analyze the frequencies of responses within each category, as it is well-suited for categorical data analysis. We acknowledge that treating ordinal data categorically rather than numerically may limit certain analytical insights, such as detecting subtle trends. Nevertheless, this categorical approach was intentionally chosen because it clearly highlights overall shifts in response distributions between the two surveyed periods, aligning with the primary objective of our analysis. The p-value was calculated to determine statistical significance, with a threshold of p < 0.05 used to indicate significance. All analyses were performed using Microsoft Excel (Microsoft® Corp., Redmond, WA). Our study encompasses three distinct sample types, illustrated in Figure 1.

General Sample of Portuguese Hospitals

Definition: Referred to as the "general sample" or "our sample," this is the primary focus of our study.

Assumption: It is presumed to be a representative sample of the general scenario in PEDs, comparing responses from 2007 and 2018.

Analysis: Data were analyzed using a chi-square test (n_2007_ = 21; n_2018_ = 23) with Yates’s continuity correction when necessary, as it is advised for analyzing a 2 × 2 contingency table, and the associated p-value was calculated.

2018-Only Sample

Scope: Analyzes responses to questions exclusively present in the 2018 questionnaire.

Exclusion: We looked only at answers by the head physician, excluding answers by nurse managers (n_2018_ = 23).

Comparison Between Head Physician and Nurse Manager Responses

Purpose: To evaluate whether physicians and nurses provided divergent responses in 2018.

Inclusion: Only responses from hospitals where both head physicians and nurse managers responded were included.

Analysis: Data were analyzed using a chi-square statistical test with Yates’s correction (n_physician+nurse_ = 13).

When answers were originally formatted as “never,” “<50% of the time,” “>50% of the time,” and “always,” we grouped hospitals in which the answer was “never” or “<50% of the time.” In some questions, we grouped hospitals into smaller groups, as described in the tables of results, with Yates’s correction, so we could define a statistical relationship with the chi-square test.

## Results

Sample

In 2007, we received responses from 21 head physicians, each representing a different hospital. In 2018, there were a total of 40 responses: 23 from head physicians and 17 from nurse managers. Among these, 13 hospitals provided responses from both the head physician and the nurse manager. This means that 47% (21 out of 45) of the Portuguese PEDs responded in 2007 and 60% (27 out of 45) in 2018. Table 1 shows the characterization of the responses as well as the medical specialties operating in different PEDs. The annual emergency episodes reported were, on average per hospital, 31,910 cases (SD = 11,286.9) in 2007 and 40,943 cases (SD = 14,737.9) in 2018.

**Table 1:** Characterization of the responses to questionnaires from Portuguese PEDs responsible staff in 2007 and 2018 PED - pediatric emergency department

Hospital nature				PED’s responsible				Medical specialties in PED							
Public (n)		Private (n)		Physician (n)		Nurse (n)		Pediatricians (n)		Pediatric Residents (n)		General practitioners (n)		Others (n)	
2007	2018	2007	2018	2007	2018	2007	2018	2007	2018	2007	2018	2007	2018	2007	2018
21	37	0	3	21	23	0	17	20	39	18	34	20	31	1	12

Pain protocol

A significant increase was observed in the adoption of pain management protocols by hospitals, from 52% (n = 11) in 2007 to 93% (n = 25) in 2018 (p = 0.03, chi-square with Yates’s correction). However, in 2018, among the hospitals where both nurse managers and head physicians responded, 15% (n = 2) revealed discrepancies between the professionals, with the former being unaware of the existence of such protocols (p = 0.14). In 2018, among the 25 hospitals reporting the adoption of a protocol, the average implementation occurred eight years prior to the questionnaire (SD = 5.2 years). Of the 27 hospitals sampled in 2018, 81% (n = 22) confirmed performing analgesia in triage by protocol, with no observed discrepancies in responses between nurses and physicians from the same hospitals.

Pain assessment

Prevalence of Pain Assessment

In 2007, 57% (n = 12) of hospitals reported an assessment of pain in at least 50% of emergency episodes; in 2018, all hospitals did so (p < 0,001, Z-score statistic).

Pain Assessment: Who, Method, Pain Scales, and Locus

From 2007 to 2018, our sample revealed a statistically significant increase in nurses conducting pain assessments, rising from 52% (n = 11) to 100% (n = 27) (p = 0.008). Conversely, physicians' contributions to pain assessments remained stable, with 57% (n = 12) in 2007 and 50% (n = 13) in 2018 (p = 0.94, chi-square with Yates’s correction).

In our sample, hospitals started adopting significantly more pain scales from 2007 to 2018, 62% (n = 13) versus 100% (n = 27) (p = 0.03, chi-square with Yates’s correction). The pain scales used the most, by decreasing order reported in 2018, were as follows: Numeric (n = 15); Faces (n = 14); Face, Legs, Activity, Cry, Consolability (FLACC) (n = 9), Analogic (n = 5), Neonatal Infant Pain Scale (NIPS) (n = 3), Portuguese Triage Group Scale (3), Faces Pain Scale - Revised (FPS-R) (n = 3), and Face, Legs, Activity, Cry, Consolability - Revised (FLACC-R) (n = 1).

Analgesia for different types and presentations of pain

The characterization of how pain is treated for different types of pain can be found in Table 2.

**Table 2:** Characterization of pain treatment for different types of pain concerning the general sample in 2007 and 2018: percentage (and frequency) of hospitals in which pain is or is not treated The mean of a scale from 0 to 3, in which 0 means never treated, 1 means <50% of the time, 2 means >50% of the time, and 3 means always; strong nonsteroidal anti-inflammatory drug (NSAIDs) (like diclofenac, ketorolac and metamizole)/weak opioids (like codeine and tramadol)/opioids (like morphine, fentanyl) - concerns to the percentage of hospitals in which these types of analgesics are used for pain’s treatment; χ^2^ (df) - chi-square test and degrees of freedom; p: chi-square test p-value; effect size - calculated 𝜙 when two options and Cramér's V when three options.

		General sample				
		2007	2018	χ^2^ (df)	p	Effect size
Mild to moderate pain	Never or <50% of the time	42.8% (9)	8.7% (2)	14.43 (2)	<0.01	0.57
	>50% of the time	14.4% (3)	69.6% (16)			
	Always	42.8% (9)	21.7% (5)			
	Strong NSAIDs	0% (0)	36% (8)	6.6 (1)	0.01	0.39
	Weak opioids	4.8% (1)	14% (3)	0.14 (1)	0.71	0.05
Severe pain	Not always	4.8% (1)	13% (3)	2.16 (1)	0.34	0.22
	Always	95.2% (20)	87% (20)			
	Strong NSAIDs	4.8% (1)	56.5% (13)	13.55 (1)	<0.01	0.56
	Opioids	86% (18)	93% (21)	0.01 (1)	0.91	0.02
Abdominal pain	Never or <50% of the time	76.2% (16)	47.8% (11)	6.38 (2)	0.04	0.38
	>50% of the time	9.5% (2)	43.5% (12)			
	Always	14.3% (3)	8.7% (2)			
	Strong NSAIDs	0% (0)	47.8% (11)	13.39 (1)	<0.01	0.55
	Opioids	24% (5)	36% (8)	0.31 (1)	0.58	0.08
Articular pain	Never or <50% of the time	33.3% (7)	8.7% (2)	14.66 (2)	<0.01	0.58
	>50% of the time	9.5% (2)	65.2% (15)			
	Always	57.2% (12)	26.1% (6)			
	Strong NSAIDs	4.8% (1)	47.8% (11)	4.49 (1)	<0.01	0.32
	Opioids	14.3% (3)	43.5% (10)	4.70 (1)	0.03	0.33
Fracture pain	Not always	14.3% (3)	43.5% (10)	4.49 (1)	0.03	0.32
	Always	85.7% (18)	56.5% (13)			
	Strong NSAIDs	28.6% (6)	47.8% (11)	3.49 (1)	0.06	0.28
	Opioids	52% (11)	79% (18)	0.77 (1)	0.38	0.13
Burn pain	Not always	9.5% (2)	39.1% (9)	5.13 (1)	0.02	0.34
	Always	90.5% (19)	60.9% (14)			
	Strong NSAIDs	28.6% (6)	56.5% (13)	3.49 (1)	0.06	0.28
	Opioids	52% (11)	79% (18)	0.01 (1)	0.92	0.02
Polytrauma pain	Never or <50% of the time	19% (4)	4.3% (1)	12.60 (2)	<0.01	0.54
	>50% of the time	0% (0)	43.5% (10)			
	Always	81% (17)	52.2% (12)			
	Strong NSAIDs	61.9% (13)	79.2% (18)	2.37 (1)	0.12	0.23
	Opioids	66% (14)	100% (23)	2.55 (1)	0.11	0.24

Analgesia for Mild-Moderate and Severe Pain

In our sample, the practice of using analgesia for mild to moderate pain in at least 50% of the time witnessed an increase from 57% (n = 12) to 91% (n = 21) (p = 0.0007, Z-score). Conversely, always using analgesia for mild to moderate pain experienced a significant decline from 43% (n = 9) to 22% (n = 5) (p = 0.005, Z-score). Regarding severe pain, no significant statistical difference was observed between the years, as 95% (n = 20) of the sample consistently used analgesia in such scenarios in 2007, compared to 87% (n = 20) in 2018 (p = 0.340). There is a very significant increase in the use of strong nonsteroidal anti-inflammatory drugs (NSAIDs) like diclofenac, ketorolac, and metamizole from 2007 to 2018 for mild-moderate and severe pain across our sample (p = 0.014 and p = 0.0002, respectively). For severe pain, there is a widespread use of opioids in these situations, both in 2007 and 2018.

Analgesia for Abdominal, Articular, Fracture, Burn, and Polytrauma Pains

In our sample, a significant increase was observed in the number of hospitals that use analgesics (either more than 50% of the time or always) in patients with abdominal pain, from 24% (n = 5) in 2007 to 52% (n = 14) in 2018 (p = 0.041). For the same question, but concerning articular pain, there was also a significant increase, from 67% (n = 13) in 2007 to 91% (n = 21) in 2018 (p = 0.006). In both scenarios, there is a significant increase in the use of strong NSAIDs from 2007 to 2018 (p = 0.0003 and p = 0.0014, respectively). Regarding articular pain, we could also find a significant increase in the use of opioids, from 14% (n = 3) in 2007 to 44% (n = 10) in 2018 (p = 0.03).

There was a non-statistically significant evolution of use of analgesics for fracture pain and burn, with a similar use of analgesia in those cases in both years. In these cases, there is a widespread use of strong NSAIDs and opioids, but with no statistically significant difference between the years.

In our sample for polytrauma treatment, there was a statistically significant increase in the proportion of hospitals that use analgesics (either more than 50% of the time or always), from 81% (n = 17) in 2007 to 96% (n = 22) in 2018 (p = 0.002).

Procedural analgesia and sedation

Characterization of different procedures of sedoanalgesia can be found in Table 3.

**Table 3:** Characterization of pain treatment of different procedures concerning the general sample in 2007 and 2018: percentage number (and frequency) of hospitals in which pain is or is not treated The mean of a scale from 0 to 2, in which 0 means never treated, 1 means <50% of the time and 2 means >50% of the time (except for local analgesia for intramuscular injections which was closed question, answering yes/no); χ^2^ (df) - chi-square test and degrees of freedom; p: chi-square test p-value; effect size - calculated 𝜙 when two options and Cramér's V when three options.

		General sample				
		2007	2018	χ^2^(df)	p	Effect size
Venoanalgesia	Never	81% (17)	82.6% (19)	2.04 (2)	0.36	0.21
	<50% of the time	4.8% (1)	13% (3)			
	>50% of the time	14.2% (3)	4.4% (1)			
Local analgesia for intramuscular injections	Yes	57.1% (12)	65.2% (15)	0.30 (1)	0.58	0.08
	No	42.9% (11)	34.8% (8)			
Analgesia for lumbar puncture	<50% of the time	76.2% (16)	78.3% (18)	0.03 (1)	0.87	0.02
	>50% of the time	23.8% (5)	21.7% (5)			
Sedation for lumbar puncture	<50% of the time	100% (21)	91.3% (21)	1.91 (1)	0.17	0.21
	>50% of the time	0% (0)	8.7% (2)			
Topical analgesia for lumbar puncture	Yes	52.4% (11)	73.9% (17)	2.20 (1)	0.14	0.22
	No	47.6% (10)	26.1% (6)			
Local analgesia for lumbar puncture	Yes	19% (4)	0% (0)	4.82 (1)	0.03	0.33
	No	81% (17)	100% (23)			
Analgesia for fracture reduction	Never	33.3% (7)	4.4% (1)	6.42 (2)	0.04	0.38
	<50% of the time	19% (4)	34.8% (8)			
	>50% of the time	47.7% (10)	60.8% (14)			
Sedation for fracture reduction	Never	33.3% (79	4.4% (1)	16.78 (2)	<0.01	0.62
	<50% of the time	19% (4)	34.8% (8)			
	>50% of the time	47.7% (10)	60.8% (14)			
Analgesia for wound disinfection and suture	Never	19% (4)	0% (0)	12.14 (2)	<0.01	0.53
	<50% of the time	14.3% (3)	60.8% (14)			
	>50% of the time	66.7% (14)	39.2% (9)			
Sedation for wound disinfection and suture	Never	71.4% (15)	21.7% (5)	10.93 (2)	<0.01	0.50
	<50% of the time	23.8% (5)	65.2% (15)			
	>50% of the time	4.8% (1)	13.1% (3)			

Characterization of the places where the procedures of sedoanalgesia are performed can be found in Table 4.

**Table 4:** Characterization of the settings where sedoanalgesia procedures are performed, based on the general sample, including the percentage (and frequency) of hospitals where pain is treated or not treated in the specific emergency department and in the pediatric emergency department These questions were not included in the 2007 questionnaire.

			General sample	
			2007	2018
Fracture reduction	Orthopedic emergency department	Yes	NA	34.7% (8)
		No	NA	65.3% (15)
	Pediatric emergency department	Yes	NA	47.8% (11)
		No	NA	52.2% (12)
Wound disinfection and suture	Surgical emergency department	Yes	NA	69.6% (16)
		No	NA	30.4% (7)
	Pediatric emergency department	Yes	NA	69.6% (16)
		No	NA	30.4% (7)

Characterization of the use of sucrose, lidocaine/prilocaine patches (EMLA®), and equimolar mixture of oxygen and nitrous oxide (EMONO) in the procedures of sedoanalgesia can be found in Table 5.

**Table 5:** Characterization of the use of sucrose, lidocaine/prilocaine patches (EMLA®), and equimolar mixture of oxygen and nitrous oxide (EMONO) in the procedures of sedoanalgesia, including the percentage (and frequency) of hospitals where pain is treated or not treated EMLA® and EMONO questions were not included in the 2007 questionnaire; χ^2^ (df) - chi-square test and degrees of freedom; p: chi-square test p-value; effect size - calculated 𝜙.

		General sample				
		2007	2018	χ^2^ (df)	p	Effect size
Sucrose	Yes	52.4% (11)	95.6% (22)	10.96	<0.01	0.50
	No	47.6% (10)	4.4% (1)			
EMLA®	Yes	NA	85% (20)	NA	NA	NA
	No	NA	15% (3)			
EMONO	Yes	NA	37% (8)	NA	NA	NA
	No	NA	63% (15)			

Analgesia for Venopuncture and Use of Lidocaine for Intramuscular Injections

From 2007 to 2018, there was no statistically significant change in the number of hospitals that never use analgesia or use it less than 50% of the time in our sample, 86% (n = 18) and 95% (n = 21), respectively (p = 0.363).

There was also no statistically significant change in the use of local lidocaine with intramuscular injections in our sample, 57% (n = 12) in 2007 and 65% (n = 15) in 2018 (p = 0.58, chi-square with Yates’s correction). In 2018, 46% (n = 11) of the physicians confirmed the use of lidocaine compared to 85% (n = 11) of the nurses; however, this difference was not statistically significant (p = 0.10).

Analgesia and Sedation for Lumbar Puncture

There was no statistically significant variation in the proportion of hospitals that use analgesics for lumbar punctures less than 50% of the time. There was a statistically significant reduction in the proportion of hospitals that use local analgesics in our sample (p = 0.03), accompanied by a slight increase in the use of topical analgesics and sedation, but neither showed any statistically significant differences.

Analgesia and Sedation for Fracture Reduction

Regarding the use of analgesia and sedation for fracture reduction, there was a statistically significant difference in the proportion of hospitals that never used these techniques in our sample, decreasing from 33% (n = 7) in 2007 to 4% (n = 1) in 2018 (p = 0.04). This indicates an increased adoption of pain management treatments for these scenarios. According to the hospitals that answered in 2018, fracture reduction procedures were performed within PEDs in 48% (n = 11).

Analgesia and Sedation for Wound Suture

For the use of analgesia and sedation for wound disinfection and suture, there was also a statistically significant reduction in the number of hospitals that never use these techniques in our sample (p = 0.002 and p = 0.004, respectively), which also indicates an increased adoption of pain management treatments for these scenarios. Among the hospitals interviewed in 2018, 70% (n = 16) performed the wound suturing and disinfection procedure in the PED.

Use of Sucrose, Lidocaine/Prilocaine Patches (EMLA®), and EMONO

There was a statistically significant increase in terms of the use of sucrose for infants <6 months old for painful procedures in our sample from 52% (n = 11) of hospitals in 2007 to 96% (n = 22) in 2018 (p = 0.0009, chi-square with Yates’s correction). Among the hospitals interviewed in 2018, 85% (n = 20) use EMLA® and 37% (n = 8) had EMONO.

Medical Responsibility for IV Procedural Sedoanalgesia and Pre-procedural Risk Evaluation

In 2018, intensivists or anesthetists supported the sedation prescription in 85% (n = 20) of hospitals, and this prescription was performed exclusively by these physicians in still 7% (n = 2) of hospitals. The pre-sedation risk evaluation is performed in 93% (n = 21) of hospitals, and 89% (n = 20) of hospitals registered this risk and the sedation procedure.

Pain reassessment after analgesia

Concerning if efficacy of pain treatment is assessed, the results were already high in 2007, but there was a slight increase across the general sample, 81% (n = 17) in 2007 to 89% (n = 20) in 2018, yet such an increase is not statistically significant (p = 0.317, chi-square with Yates’s correction).

Non-pharmacological interventions

The increase in use of non-pharmacological interventions in our sample from 52% (n = 11) in 2007 to 83% (n = 19) in 2018 was not statistically significant (p = 0.031, chi-square with Yates’s correction). In 2018, there was no statistically significant difference between the answers provided by physicians and nurses within the same hospital concerning the use of these techniques (p = 1.00).

Among hospitals interviewed in 2018, caregivers are always or more than 50% of the time present during procedures in 93% (n = 21) of hospitals (Table 6). However, only 52% (n = 12) of hospitals confirmed using the caregiver’s lap always or more than 50% of the time. In 2018, 26% (n = 6) of hospitals reported using forced immobilization more than 50% of the time.

**Table 6:** Characterization of the percentage (and frequency) of hospitals in which caregiver presence, caregiver's lap, and forced immobilization are used in 2018

	Always	>50% of the time	<50% of the time	Never
Caregiver presence	37% (8)	56% (13)	7% (2)	0% (0)
Caregiver’s lap	7% (2)	44% (10)	30% (7)	18% (4)
Forced immobilization	0% (0)	26% (6)	70% (16)	4% (1)

General use of opioids

The general use of opioids did not change significantly both across the general sample (p = 0.94, using a chi-square with Yates’s correction). Similarly, no significant variations were observed when weak and strong opioids were compared alone. Weak opioids decreased from 48% (n = 10) in 2007 to 36% (n = 8) of hospitals in 2018 (p = 0.73, chi-square with Yates’s correction). Strong opioids had a non-statistically significant marginal increase from 61% (n = 13) to 64% (n = 15) of hospitals (p = 0.83, chi-square with Yates’s correction).

Training and adequacy of pain treatment

Staff’s Perception of Pain Treatment Adequacy and Need for Training

Concerning the perception of adequacy of pain treatment practiced in our sample, there seems to be a slight decrease from 67% (n = 14) in 2007 to 65% (n = 15) in 2018. However, these results are not statistically significant (p = 0.919, chi-square with Yates’s correction). At the same time, in our sample, there seems to be a slight increase in the will for additional training, from 86% (n = 18) in 2007 to 88% (n = 20) in 2018, also not statistically significant (p = 0.904, chi-square with Yates’s correction).

Staff’s Perception of the Main Reasons for Pain Treatment Inadequacy

The main reasons appointed for an inadequacy of pain treatment in 2007 were, from the most to the less chosen, fear of hiding symptoms or signs (38% (n = 8) of hospitals), difficulty of a correct pain assessment (33%, n = 7) and fear of opioid side effects (24%, n = 5). In 2018, the most chosen reasons were fear of hiding symptoms or signs (50% (n = 11) of hospitals), lack of time (33%, n = 8), fear of opioid side effects (29%, n = 7), and medical inexperience in prescribing analgesics (29%, n = 7).

## Discussion

From 2007 to 2018, while significant enhancements in pain assessment were observed in Portuguese PEDs, substantial opportunities remain to improve the routine implementation of pain treatment protocols and present them to all professionals of the department. Over the years, the management of severe pain and the associated use of opioids have remained satisfactory, and there has been an improvement in addressing mild to moderate pain, with a higher frequency of analgesic use in these cases. However, although the use of sedation increased significantly for many painful procedures, analgesia during these procedures persists being scarce. In line with these results, despite the improvements, there is still a considerable proportion of hospitals that acknowledge inadequate treatment of pain. Fear of children hiding symptoms/signs and hesitancy concerning opioid use account for the most prevailing reasons for unsatisfactory treatment, mirroring previously reported results [21,22]. In the other direction, the use of non-pharmacological techniques for pain control seems to have improved, with multiple hospitals reporting the child sitting on the caregiver’s lap during painful procedures, but others, however, still frequently use forced immobilization, a practice that is against ethical and legal considerations in health care [12,20,23]. Nevertheless, the widespread desire for further training on the treatment of pain by health practitioners offers a sign of hope for the future.

Pain assessment rose significantly, with the implementation of local protocols for pain management and use of recommended pain scales in a highly prevalent practice in Portugal (93% and 100%, respectively), reflecting the application of Portuguese and international guidelines and practices [3,6,11,17,24,25]. With the universal use of pain scales, nurses have settled as the main pain assessors, and the caregivers’ contribution has significantly fallen. In line with the evolution in Italy [26], there was a significant increment in pain assessment in Portugal from 2007 to 2018, but it has not yet been universally adopted as suggested [3,11].

In fact, analgesia in triage by protocol was not yet a generalized practice. Portugal’s 81% triage analgesia lags behind Australia’s 92%, suggesting a need for triage protocol standardization [5,26]. Similarly, analgesia for mild to moderate pain, even with a better approach in Portuguese PEDs, is still not a generalized practice, particularly when considering PEDs that always administer it, which represents only 22% of the total hospitals that responded in 2018. This presents a concerning scenario, as children frequently receive inadequate pain relief treatment [27]. However, there was a significant improvement in the administration of strong NSAIDs for this type of pain. Opioids are practically unused in this setting, despite the broad consensus for their use for moderate pain in lower doses [11,12]. Unlike previous studies [7], a high percentage of severe pain cases were treated during the years studied, accompanied by the widespread use of opioids in this context.

Concerning some specific pain treatments, the 11-year evolution has been generally satisfactory. Besides that, for instance, abdominal and articular pain remain non-universally treated, with few hospitals reporting treatment in all instances (17.4% on average), similarly to the results from other countries [28]. Fracture-, burn-, and polytrauma-associated pains are reported to always be treated on average in 55.1% of our cohort of hospitals. Considering that these situations are typically associated with moderate to severe pain, this percentage is inconsistent with the overall response regarding the treatment of severe pain, where 87% of Portuguese PEDs reported always using analgesia in these situations, which may reflect reluctance to escalate analgesia for procedural pain. Alongside, the generalized use of opioids has been reported for polytrauma-associated pain (100%), but less for fracture- and burn-associated pains (79% for both).

Beyond sparse adherence to specific pain treatment with analgesics, the frequency of analgesia for various medical procedures remains uncommon, and incorrect practices continue. Notably, there is a slight reduction in hospitals that never use analgesics for these procedures, but analgesia remains rarely used for venipuncture (83% of the hospitals in 2018 never use), below the average in Europe and Canada [29], but in line with other international reports [28]. National and international recommendations, however, clearly suggest the implementation of analgesia when performing venipuncture [11,12,20,23]. On the other hand, sucrose is now widely used in Portuguese PEDs, contrasting with other nations where this practice is less frequent [28]. Sedation for painful procedures incremented considerably and, in fact, only a few hospitals (13.1% on average) never used sedation during these practices, despite EMONO unavailability in most Portuguese PEDs. Indeed, the availability of EMONO is below the European mean [29]. In a few Portuguese PEDs, the wrong practice of limiting opioid and sedation prescription to intensivists/anesthetists persists (7% for both) [11,30]. This reflects the reduced specific training of general pediatricians on procedural analgesia and sedation and the willingness for additional learning. 

About our statistical analysis, while we employed the chi-square test to assess associations between categorical variables, we acknowledge its limitations. The test is sensitive to sample size and is more robust with larger samples; our relatively small sample (yet, always >5) may limit generalizability and reduce the power to detect subtle associations. Additionally, the chi-square test identifies the presence of statistical associations but does not provide information about their strength or direction, necessitating cautious interpretation. Finally, the test is designed for categorical data; it treats Likert scale responses as nominal rather than ordinal, potentially overlooking nuanced trends inherent in the data's ordinal nature.

Our study has some limitations including (1) the comparison of two different populations, since we compared the answers from 2007 with the ones from 2018, and not only the same hospitals; (2) the absence of control for non-response biases, i.e., the hospitals that responded to questionnaires probably had more interest in this subject; (3) the responses of the head physicians and nurse managers can be biased by different emphasis in the course of their education; (4) the sample size despite representing 60% of Portuguese PEDs is still statistically small, some results would have improved statistical power given larger samples; (5) non-pharmacological techniques were explored less objectively than pharmacological techniques, limiting the evaluation of the desired multimodal approach to pediatric pain [19]. Despite these limitations, our cross-sectional study offers an insightful view over a decade of pain assessment and treatment at a countrywide level, allowing the definition of a clear roadmap to improve the quality of treatment in Portuguese PEDs.

## Conclusions

In recent years, Portugal has made significant progress in the assessment and management of pediatric pain, with the implementation of local protocols and the widespread adoption of pain scales marking important steps forward. Nonetheless, challenges persist, particularly in the consistent treatment of mild to moderate pain and the limited use of analgesia in procedural contexts. While the management of severe pain and sedation practices has improved, these strategies are not yet universally applied. A coordinated national strategy is urgently needed, emphasizing knowledge translation, targeted local training, and optimal use of existing resources. Finally, robust scientific research, regular system evaluations, and international collaborations are essential to sustain and deepen progress in this field.

## Appendices

Questionnaire - Management of pediatric pain in the pediatric emergency department

Dear colleague,

We ask for 10 minutes of your time to answer this questionnaire.

We have the goal of exploring the actual practices of pain management in national pediatric emergency departments. To do so, I send you this questionnaire, which I would like you to respond to accordingly.

This questionnaire was already sent in 2007, and the results were presented in March of the same year at the second meeting of the pediatric department of Hospital Professor Dr. Fernando Fonseca, EPE.

Your participation is totally voluntary and without any kind of prejudice if you decide not to participate. The information shared will be protected and exclusively accessed by the team from the study. Every professional on this team is restricted by professional secrecy.

Thank you for your time. Please, if you have any questions or need any additional information, feel free to contact us:

André Garrido - Pediatric Resident - andre.garrido@hff.min-saude.pt Clara Abadesso - Pediatrician - clara.s.abadesso@hff.min-saude.pt 
 

Email

____________________________________________

What is your role in the pediatric emergency department (PED)?

☐Physician - the head physician

☐Nurse - the nurse manager

Which is your hospital?

___________________________________________

Hospital type:

☐General hospital

☐Pediatric hospital

Average number of PED visits:

_______________________

Which type of physicians work at your PED?

☐Pediatricians

☐Pediatric residents

☐General practitioner

☐Others

Pain Protocol and Assessment

Is there a protocol for pain management at your PED?

☐Yes

☐No

If yes, for how long?

______________________

Is analgesia done in triage?

☐Yes

☐No

How frequently is pain assessed at your PED?

☐Always

☐> 50% of the time

☐< 50% of the time

☐Never

Who assesses pain?

☐The nurse from triage

☐The physician

How is it done?

☐Nurse’s impression

☐Physician’s impression

☐ Parents’ impression

☐Pain assessment scale

☐Other

Which pain assessment scales are used?

______________________________________________________________________

Is pain reassessed after analgesia?

☐Yes

☐No

Analgesia for Different Types and Presentations of Pain

How frequently is analgesia done for mild to moderate pain?

☐Always

☐> 50% of the time

☐< 50% of the time

☐Never

Which drugs are used to do it?

☐Paracetamol

☐Ibuprofen

☐Diclofenac

☐Ketorolac

☐Dipyrone

☐Other NSAIDs

☐Tramadol

☐Morphine

☐Fentanyl

☐Other opioids

☐Other

How frequently is analgesia done for severe pain?

☐Always

☐> 50% of the time

☐< 50% of the time

☐Never

Which drugs are used to do it?

☐Paracetamol

☐Ibuprofen

☐Diclofenac

☐Ketorolac

☐Dipyrone

☐Other NSAIDs

☐Tramadol

☐Morphine

☐Fentanyl

☐Other opioids

☐Other

How frequently is analgesia done for abdominal pain?

☐Always

☐> 50% of the time

☐< 50% of the time

☐Never

Which drugs are used?

☐Paracetamol

☐Ibuprofen

☐Diclofenac

☐Ketorolac

☐Dipyrone

☐Other NSAIDs

☐Tramadol

☐Morphine

☐Fentanyl

☐Other opioids

☐Other

How frequently is analgesia done for articular pain?

☐Always

☐> 50% of the time

☐< 50% of the time

☐Never

Which drugs are used?

☐Paracetamol

☐Ibuprofen

☐Diclofenac

☐Ketorolac

☐Dipyrone

☐Other NSAIDs

☐Tramadol

☐Morphine

☐Fentanyl

☐Other opioids

☐Other

How frequently is analgesia done for bone fracture-associated pain?

☐Always

☐> 50% of the time

☐< 50% of the time

☐Never

Which drugs are used?

☐Paracetamol

☐Ibuprofen

☐Diclofenac

☐Ketorolac

☐Dipyrone

☐Other NSAIDs

☐Tramadol

☐Morphine

☐Fentanyl

☐Other opioids

☐Other

How frequently is analgesia done for polytrauma-associated pain?

☐Always

☐> 50% of the time

☐< 50% of the time

☐Never

Which drugs are used?

☐Paracetamol

☐Ibuprofen

☐Diclofenac

☐Ketorolac

☐Dipyrone

☐Other NSAIDs

☐Tramadol

☐Morphine

☐Fentanyl

☐Other opioids

☐Other

How frequently is analgesia done for burn-associated pain?

☐Always

☐> 50% of the time

☐< 50% of the time

☐Never

Which drugs are used?

☐Paracetamol

☐Ibuprofen

☐Diclofenac

☐Ketorolac

☐Dipyrone

☐Other NSAIDs

☐Tramadol

☐Morphine

☐Fentanyl

☐Other opioids

☐Other

Procedural Analgesia and Sedation

Is sucrose used for painful procedures in infants <6 months old?

☐Yes

☐No

Is EMONO used for procedural sedoanalgesia?

☐Yes

☐No

Is EMLA® used for procedural analgesia?

☐Yes

☐No

Is local analgesia done for intramuscular injections?

☐Yes

☐No

How frequently is analgesia done for venopuncture?

☐Always

☐> 50% of the time

☐< 50% of the time

☐Never

What kind of analgesia is used in these situations?

________________________________________________________________

How frequently is analgesia done for lumbar puncture?

☐Always

☐> 50% of the time

☐< 50% of the time

☐Never

What kind of analgesia is used in these situations?

________________________________________________________________

How frequently is sedation done for lumbar puncture?

☐Always

☐> 50% of the time

☐< 50% of the time

☐Never

Which drugs do you use for lumbar puncture sedations?

________________________________________________________________

How frequently is analgesia done for fracture reductions?

☐Always

☐> 50% of the time

☐< 50% of the time

☐Never

What kind of analgesia is used in these situations?

________________________________________________________________

How frequently is sedation done for fracture reductions?

☐Always

☐> 50% of the time

☐< 50% of the time

☐Never

Which drugs do you use for these sedations?

________________________________________________________________

Where are fracture reductions done?

☐Orthopedic emergency department

☐PED

☐Other

How frequently is analgesia done for wound disinfection and suture?

☐Always

☐> 50% of the time

☐< 50% of the time

☐Never

What kind of analgesia is used in these situations?

________________________________________________________________

How frequently is sedation done for wound disinfection and suture?

☐Always

☐> 50% of the time

☐< 50% of the time

☐Never

Which drugs do you use for these sedations?

________________________________________________________________

Where are wound disinfections and sutures done?

☐Surgery emergency department

☐PED

☐Other

Who is responsible for the IV procedural sedoanalgesia?

☐Pediatrician

☐Anesthetists

☐Any physician

Is there support for intensivists or anesthetists for moderate to profound sedation?

☐Yes

☐No

Is pre-sedation risk evaluated?

☐Yes

☐No

Is pre-sedation risk, monitoring, and drugs used registered after the procedure?

☐Yes

☐No

General Use of Opioids

Opioid prescription is done by:

☐Any physician of the PED

☐Only pediatricians

☐Only with the support of the intensivists or anesthetists

☐Other

Non-Pharmacological Interventions

Are non-pharmacological interventions used?

☐Yes

☐No

How frequently is forced immobilization used?

☐Always

☐> 50% of the time

☐< 50% of the time

☐Never

How frequently are parents present for procedures?

☐Always

☐> 50% of the time

☐< 50% of the time

☐Never

How frequently is the parent’s lap used?

☐Always

☐> 50% of the time

☐< 50% of the time

☐Never

Training and Adequacy of Pain Treatment

Do you think that pain is adequately managed at your PED?

☐Yes

☐No

If you do not think so, why?

☐Difficulty in communicating with children

☐ Difficulty of a correct pain assessment

☐Inadequate methods

☐Lack of time

☐Lack of motivation

☐Lack of material

☐Inadequate medical prescriptions

☐Medical inexperience in prescribing analgesics

☐Fear of hiding symptoms and signs

☐Fear of opioid side effects

☐Insufficient child and parents’ information

☐Insufficient staff knowledge of how to manage pain

☐Procedures are too quick, they don’t need analgesia

☐Pain is natural, we should not treat it every time

☐Other

If you responded “other”, please specify it

___________________________________________________________________

Do you think that your staff should have more training of in pain management?

☐Yes

☐No

Comments

___________________________________________________________________
